# Secondary Highly Pathogenic Porcine Reproductive and Respiratory Syndrome Virus (HP-PRRSV2) Infection Augments Inflammatory Responses, Clinical Outcomes, and Pathogen Load in *Glaesserella-parasuis*-Infected Piglets

**DOI:** 10.3390/vetsci10050365

**Published:** 2023-05-20

**Authors:** Zhixin Guan, Linlin Pang, Yan Ouyang, Yifeng Jiang, Junjie Zhang, Yafeng Qiu, Zongjie Li, Beibei Li, Ke Liu, Donghua Shao, Zhiyong Ma, Jianchao Wei

**Affiliations:** 1 Shanghai Veterinary Research Institute, Chinese Academy of Agricultural Science, No. 518, Ziyue Road, Shanghai 200241, China; 2 College of Agriculture, Hubei Three Gorges Polytechnic, Yichang 443000, China

**Keywords:** *Glaesserella parasuis*, HP-PRRSV2, pork industry, inflammatory cytokines

## Abstract

**Simple Summary:**

Based on the fact that Gps are rooted in the upper respiratory tract of pigs, in order to investigate whether secondary infection with HP-PRRSV can exacerbate lung disease and chronic inflammation, our study was designed as follows. Our study randomly divided piglets into four groups: Gps + HP-PRRSV, Gps, HP-PRRSV, and controls. Piglets in the Gps + HP-PRRSV and Gps groups were infected through the intranasal route with the Gps W2 strain. The Gps + HP-PRRSV and HP-PRRSV groups were challenged with the HP-PRRSV HuN4 strain by intramuscular injection and intranasally at 5 days after the initial exposure to Gps. Alternatively, the control group animals received phosphate-buffered saline (PBS). Then, we observed the influence of HP-PRRSV–Gps coinfection on clinical outcomes, pathogen shedding and loading, cytokine production, and specific antibody levels at various time points in infected piglets. Our results revealed synergistic effects in HP-PRRSV–Gps coinfection, which increase the severity of clinical signs compared with single infections. Therefore, in the unavoidable situation of Gps infection in piglets, necessary measures must be made to prevent and control secondary infection of HP-PRRSV, which can save huge economic losses to the pork industry.

**Abstract:**

*Glaesserella parasuis* (Gps), Gram-negative bacteria, are a universal respiratory-disease-causing pathogen in swine that colonize the upper respiratory tract. Highly Pathogenic Porcine Reproductive and Respiratory Syndrome Virus (HP-PRRSV2HP-PRRSV2) and Gps coinfections are epidemics in China, but little is known about the influence of concurrent coinfection on disease severity and inflammatory responses. Herein, we studied the effects of secondary HP-PRRS infection on clinical symptoms, pathological changes, pathogen load, and inflammatory response of Gps coinfection in the upper respiratory tract of piglets. All coinfected piglets (HP-PRRSV2 + Gps) displayed fever and severe lesions in the lungs, while fever was present in only a few animals with a single infection (HP-PRRSV2 or Gps). Additionally, HP-PRRSV2 and Gps loading in nasal swabs and blood and lung tissue samples was significantly increased in the coinfected group. Necropsy data showed that coinfected piglets suffered from severe lung damage and had significantly higher antibody titers of HP-PRRSV2 or Gps than single-infected piglets. Moreover, the serum and lung concentrations of inflammatory cytokines (TNF-α, IL-1β, IL-6, and IL-8) were also significantly higher in coinfected piglets than in those infected with HP-PRRSV2 or Gps alone. In conclusion, our results show that HP-PRRSV2 promotes the shedding and replication of Gps, and their coinfection in the upper respiratory tract aggravates the clinical symptoms and inflammatory responses, causing lung damage. Therefore, in the unavoidable situation of Gps infection in piglets, necessary measures must be made to prevent and control secondary infection with HP-PRRSV2, which can save huge economic losses to the pork industry.

## 1. Introduction

China is the largest pork-producing country [1]. Porcine Respiratory Disease Complex (PRDC) is the most challenging health concern for pig production worldwide, including in China [2]. PRDC often involves coinfections of viruses, such as swine Influenzas A Virus (swIAV) [3], PRRSV, and Porcine Circo Virus type 2 (PCV2), with bacteria such as *Actinobacillus pleuropneumonia* (APP), *Mycoplasma hyopneumoniae* (Mh), and *Bordetella bronchiseptica* (Bb) [4]. PRRSV can cause coinfection or secondary infection with bacteria such as Gps, *Streptococcus suis* (SS), and APP [3,5,6,7]. Notably, PRRSV outbreaks in July 2006 caused huge economic losses to the pork industry in China [8,9,10]. Such outbreaks can cause 100% morbidity and >40% mortality in infected piglets [11,12]. In PRRSV-infected animals, the respiratory tract becomes more prone to other pathogens, such as PCV2 [13,14,15], Classical swine fever virus (CSFV), SS, Gps, Mh, and various *Salmonella species* (spp), which may affect the severity of PRRSV-induced pathogenesis [16,17,18,19,20,21]. Previous studies on bacterial ecology in Chinese pig farms identified SS (77.92%), Gps (51.25%), *Escherichia coli* (52.39%), and *Pasteurella multicide* (26.77%) infections, along with the endemic of PRRSV, under field conditions [1,20,22]. Interestingly, studies comparing pathogens in healthy pigs with those with pneumonia found that PRRSV, PCV2, and Gps were prevalent among all samples regardless of the presence/absence of related clinical symptoms [23,24,25]. Overall, these data indicated that PRRSV and bacterial coinfections have high occurrences in the Chinese pig population [7]. In most cases, multiple infectious agents involved in the development of clinical conditions make the universal reductionist approach impractical to examine the host–pathogen interactions, as in the case of single infections [20].

Gps is a Gram-negative, non-hemolytic, nicotinamide adenine dinucleotide (NAD) dependent bacterium [26]. Gps is not only a universal bacterium of the upper respiratory tract of pigs, but also an important universal respiratory pathogen causing fibrinous polyserositis, arthritis, and meningitis in pigs [27,28,29,30]. Gps infections lead to high mortality rates in pigs, causing significant economic losses to the swine industry worldwide [31,32]. Gps strain can also be obtained from the nasal cavities, tonsils, and tracheae of healthy pigs [33,34]. Under favorable conditions, Gps activates innate immune responses promoting the production of inflammatory cytokines [35]. Interestingly, PRRSV infection can have an additive effect on Gps infection and loading [33]. However, the factors causing Gps systemic infection in pigs have not yet been identified [36].

The PRRSV is a single-stranded positive-sense RNA virus with a capsid [37,38,39]. The PRRSV disease was first identified in the United State of America in the late 1980s and in Germany in the 1990s [40,41,42,43,44], and then it spread to other pig-producing areas, including China, causing enormous economic losses to the pig industry [45,46,47,48]. PRRSV is classified in the order *Nidovirales*, genus *Arterivirus*, family *Arteviridae*, together with *Equine Arteritis Virus* (EAV), mouse *Lactate Dehydrogenase-elevating Virus* (LDV), and *Simian Hemorrhagic Fever Virus* (SHFV) [42,49,50]. Based on genetic and antigenic determinants, PRRSV is divided into two major genotypes: PRRSV1 (the European *Lelystad* strain) and PRRSV2 (the North American Vr-2332 strain) [51,52]. Phylogenetically, PRRSV1 is further classified into three subtypes [53,54]: pan-European subtype 1, and East-European subtypes 2 and 3, while PRRSV2 is classified into nine distinct subtypes [7,55]. PRRSV replicates in monocytic lineage cell types, particularly porcine alveolar macrophages (PAMs), and the viral disease is characterized by severe interstitial pneumonia, reduced growth, and high mortality in young piglets, and reproductive failure in sows [20,56]. In China, PRRSV2 was first reported by Baoqing Guo in 1996 [57], while HP-PRRSV2 was identified by Kegong Tian from the Jiangxi Province in 2007 [58]. Then, HP-PRRSV2 spread to other pig-producing regions, causing serious pecuniary losses. The NADC30-like PRRSV2 was described by Zhao in 2015 [14,59,60]. HP-PRRS2V and NADC30-like PRRSV2 became prevalent after 2015 [20]. PRRSV1 and NADC34-like PRRSV2 were separately reported by Nanhua Chen in 2011 [61] and Hongliang Zhang in 2018 [62], which increased the difficulty of prevention and control of PRRSV in China [20].

PRRSV infection makes pigs susceptible to secondary infection by damaging PAM cells and inducing nasal mucositis [63]. For instance, a high detection rate of Gps is common in pig farms infected with PRRSV [64]. Yu et al. found that HP-PRRSV2 promotes Gps proliferation in blood and tissues, which increases the susceptibility to Gps in PRRSV-positive pigs [31]. Li et al. found that the transfection of Gps RNA enhanced HP-PRRSV2-mediated inflammatory response in coinfected animals [7]. Zhang et al. showed that the PRRSV2–Gps coinfection aggravated lung diseases and chronic inflammation by modulating host gene expression [33]. In the current trend, the high recombination rate and spread ability of PRRSV strains increase the incidence and mortality rates in Gps infections [65].

Although HP-PRRSV2–Gps coinfection is very common, little is known about the effects of coinfection on the pathogenicity, disease severity, inflammatory mediators, and antibody responses in infected piglets [66]. Studying the mechanism of coinfection can help control Gps outbreaks. Accordingly, in this study, we investigated the influence of HP-PRRSV2–Gps coinfection on clinical outcomes, pathogen shedding and loading, cytokine production, and specific antibody levels at various time points in infected piglets. Our results revealed synergistic effects in HP-PRRSV2–Gps coinfection, which increase the severity of clinical signs compared with single infections.

## 2. Materials and Methods

### 2.1. Ethics Statements

All animal experiments were carried out according to ethical guidelines and were approved by the Institutional Laboratory Animal Care and Use Committee of Shanghai Veterinary Research Institute (SHVRI) CAAS, Shanghai (IACUC no: SHVRI-P-2018010503), and performed in compliance with the Guidelines on Humane Treatment of Laboratory Animals (Ministry of Science and Technology of the People’s Republic of China, policy no. 2006 398).

### 2.2. Virus and Bacterium

The HP-PRRSV2 HuN4 strain (GenBank: EF635006) was kindly provided by Professor Guangzhi Tong (Shanghai Veterinary Research Institute, Chinese Academy of Agricultural Sciences). The virus was propagated in the MARC-145 cell line and cultured in Dulbecco’s modified eagle’s medium (DMEM; Invitrogen, Waltham, MA, USA), with 10% fetal bovine serum (FBS; Thermo Scientific, Waltham, MA, USA) and antibiotics (100 units of penicillin, 10 mg streptomycin, and 25 mg amphotericin B per mL; Sigma-Aldrich, St. Louis, MO, USA) at 37 °C and 5% CO_2_. The stock virus titer for experimental infection was 3 × 10^3^ TCID50/mL [45].

The virulent Gps W2 strain (serotype 13) was isolated from Shanghai, China, in March 2016, and confirmed by agglutination and agar diffusion tests. Gps was cultured on tryptic soy agar (TSA; Difco Laboratories, Franklin Lakes, NJ, USA) containing 10 mg/mL NAD and 5% calf bovine serum. The amount of W2 strain used for intranasal infection was 3 × 10^8^ CFU/mL [67].

### 2.3. Pigs, Experimental Design, and Sampling

Thirty-two 4-week-old castrated male DLY (Duroc  ×  Landrace  ×  Yorkshire) weaned piglets from the same commercial farm were used in this study. Before beginning the experiments, all piglets were tested for PRRSV, CSFV, PCV2, swine influenza virus (SIV), and Gps antigen by quantitative real-time PCR (qRT-PCR), and for PRRSV and Gps antibodies using commercial ELISA kits. All piglets were negative for PRRSV and Gps. After arrival at the animal house, the animals were acclimated for 7 days before starting the experiment.

Piglets were stochastically divided into four groups (*n* = 8): Gps + HP-PRRSV2, Gps, HP-PRRSV2, and control. Each group was housed in a separate room and provided with a basal diet (based on the NRC (2012) recommendation for the nutrient requirements of weaned piglets) [68] and water throughout the experiment.

Piglets in the Gps+ HP-PRRSV2 and Gps groups were infected through the intranasal route with 2.0 mL of Gps W2 strain (3 × 10^8^ CFU/mL). The Gps + HP-PRRSV2 and HP-PRRSV2 groups were challenged with 3.0 mL of HP-PRRSV2 HuN4 strain (3 × 10^3^ TCID_50_) by intramuscular injection (1 mL) and intranasally (2 mL) at 5 days after the initial exposure to Gps. Alternatively, the control group animals received phosphate-buffered saline (PBS). The dose and timing of HP-PRRSV2 inoculation were according to Yang et al. (2012).

Piglets were monitored daily for clinical signs, and rectal temperatures were recorded before feeding. At 0, 3, 6, 10, 14, 17 and 21 days post-infection (dpi), nasal swabs and blood samples were collected to determine the pathogen (HP-PRRSV2 /Gps) load, HP-PRRSV2/Gps antibody titer, and the serum levels of inflammatory cytokines. Three pigs per group were randomly euthanized at 6 days post-HP-PRRSV2 challenge, and blood, heart, liver, spleen, lung, and kidney samples were collected for analysis. The remaining pigs in each group were euthanized at 21 dpi.

### 2.4. Clinical and Pathological Evaluation

After Gps and HP-PRRSV2 challenge, piglets were monitored daily and scored for clinical signs, including mental state, rectal temperature, anorexia, dyspnea, coughing, and other symptoms, such as mortality, trembling, cyanosis, vomiting, diarrhea, and limping.

The piglets were scored according to the scoring criteria in Table 1. All scores were accumulated to give a total clinical score for each pig (0–8) [67]. For necropsy, lung sections were fixed in 10% neutrally buffered formalin for histological examination using hematoxylin and eosin (H&E) staining (Wuhan Service Biotechnology Co. Ltd., Wuhan, China), and the histopathological changes were observed under an optical microscope (Olympus, Tokyo, Japan).

### 2.5. Detection of HP-PRRSV2/Gps Antibodies by ELISA

Serum samples were detected by an enzyme-linked immunosorbent assay (ELISA) kit for PRRSV antibodies (IDEXX Laboratories, Atlanta, GA, USA) and a swine Gps ELISA kit (Biovet, Karnataka, India) for Gps antibodies, according to the manufacturers’ instructions.

### 2.6. Detection of Pathogen Load by qRT-PCR

Bacterial DNA was extracted using the DNeasy Blood and Tissue Kit (QIAGEN, Tokyo, Japan) and viral RNA was extracted with a Viral DNA/RNA Kit (OMEGA, Tokyo, Japan), according to the manufacturers’ instructions [67]. The quantity of Gps and HP-PRRSV2 in piglet samples was detected by qRT-PCR [31,44] performed on an ABI Step One thermocycler (Applied Biosystem, Waltham, MA, USA).

### 2.7. Cytokine Assays

Piglet lung tissues from necropsy were analyzed for cytokines [67]. Briefly, 1 g of lung tissue was homogenized in 1 mL of PBS (1:1, *w*/*v*) with an X620 tissue homogenizer (CAT). The homogenate was centrifuged at 3000 g for 10 min and the obtained supernatants were stored at −80 °C for cytokine analysis. Piglet serum samples were collected at 0, 3, 6, 7, 14, 17, and 21 dpi. The lung tissues and serum concentrations of inflammatory factors (IL-1β, TNF-α, IL-6, and IL-8) were quantified with respective ELISA kits (R&D Systems, Minneapolis, MN, USA), following standard protocols. The corresponding mRNA levels were determined using an ABI Step One thermocycler (Applied Biosystems) and relative gene expression levels were normalized against *β-actin* using the 2^−ΔΔCT^ method [69]. All tests were performed in triplicate.

### 2.8. Statistical Analysis

All statistical analyses were performed using Student’s *t*-tests in SPSS version 16.0 (SPSS Inc, Chicago, IL, USA). Data are presented as the mean ± standard deviation (SD), and those with *p*-value < 0.05 were considered statistically significant.

## 3. Results

### 3.1. Clinical Evaluation

**Figure 1:**
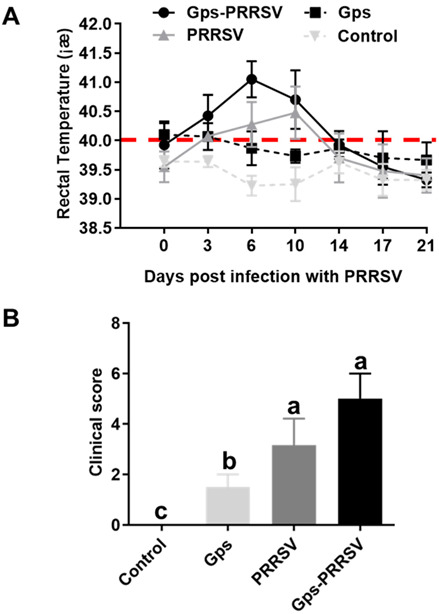
Clinical observations in piglets. (**A**) Rectal temperature (mean ± SD) and (**B**) clinical score (mean ± SD) of piglets inoculated with Gps and HP-PRRSV2 (Gps-PRRSV), Gps alone (Gps), HP-PRRSV2 alone (PRRSV), and placebo (control). The coinfected piglets were challenged with HP-PRRSV2 at 5 days post-Gps infection. After HP-PRRSV2 infection, piglets in the Gps+ HP-PRRSV2 and HP-PRRSV2 alone groups displayed significantly higher mean temperatures than those in other groups. Significant differences between the groups are marked by different superscript letters (*p* < 0.05). The red dotted line in Figure 1A is the threshold of mean rectal temperature for piglets, indicating that the temperature above the red line belongs to the range of fever, while below the red line belongs to the range of normal.

Piglets challenged with Gps alone showed mild clinical signs, while the same was absent in the control group throughout the experiment. However, the animals infected with HP-PRRSV2 alone or coinfected with Gps developed significant clinical signs including depression, drowsiness, loss of appetite, fever, anorexia, lethargy, coughing, shivering, and breathing difficulties. Among the two, the HP-PRRSV2–Gps-coinfected group was more severely affected than the HP-PRRSV2 alone group (Figure 1).

Animals inoculated with Gps alone exhibited low-grade fever at 1 and 2 dpi (temperature 40–40.5 °C), and only 50% of the animals (3/6) displayed a rise in body temperature. On the other hand, piglets in the Gps + HP-PRRSV2 and HP-PRRSV2 alone groups had fever following HP-PRRSV2 infection (rectal temperature ≥ 40 °C). Moreover, high rectal temperatures were observed in piglets exposed to HP-PRRSV2 alone from 3 to 12 dpi (Figure 1A).

HP-PRRSV2–Gps coinfection triggered major responses: the mean rectal temperature was 41 °C at 6 days after the HP-PRRSV2 challenge and remained higher than normal until 14 dpi (Figure 1A). Animals exposed to Gps alone did not show any significant rise in body temperature compared with the control group. There were significant differences among mean rectal temperatures in animals of the three infected groups. However, at 14 days after HP-PRRSV2 infection, fever disappeared in all challenged animals (Figure 1A). Clinical scores in animals were obtained by observing clinical symptoms (dyspnea, coughing, runny nose, anorexia, limping, and diarrhea). These clinical symptoms were most apparent from day 3 to day 10 in the HP-PRRSV2–Gps-coinfected group. The mean clinical scores (±SD) in all groups are shown in Figure 1B. Individual clinical scores ranged from 0 to 2 in Gps alone (1.50 ± 0.41), and 0 to 4 in the HP-PRRSV2 alone group (3.17 ± 0.73), while the same ranged from 2 to 6 in coinfected pigs (5.12 ± 0.61) (Figure 1B). Pigs in the control group did not exhibit any clinical signs. There were statistically significant differences in the mean clinical scores among the three infected groups (*p* < 0.05).

### 3.2. Pathological Examination

**Figure 2:**
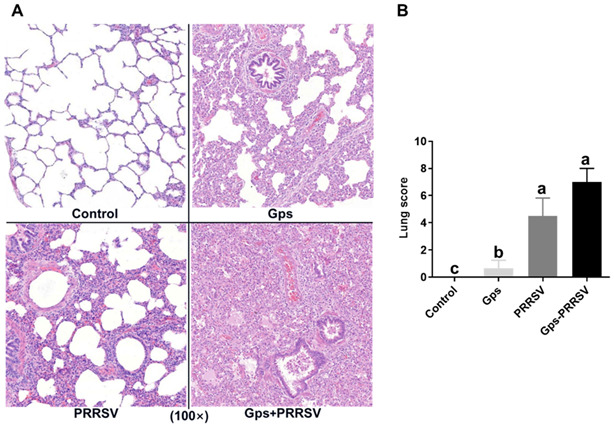
Pathological examination of the lung from all piglet groups at 6 dpi. (**A**) Histopathological changes and (**B**) the lung score (mean ± SD) of piglets inoculated with Gps and HP-PRRSV2 (Gps +PRRSV), Gps alone (Gps), HP-PRRSV2 alone (PRRSV), and placebo (control). All lung tissues were collected from each group of randomly euthanized piglets at 6 days post-HP-PRRSV2. The coinfected piglets were challenged with HP-PRRSV2 at 5 days post-Gps infection. Significant differences between the groups are marked by different superscript letters (*p* < 0.05).

We conducted post-mortem examinations at 6 dpi and the results revealed gross lesions, including lung congestion or consolidation, inguinal lymph node tumidity and hemorrhage, submandibular lymph node tumidity and hemorrhage, liver congestion tumidity, spleen infarction, kidney hemorrhage or grey spot, and brain hemorrhage or edema in the coinfected group (Figure 2A). The HP-PRRSV2 alone group also displayed pulmonary interstitial pneumonia and pulmonary alveolar epithelial damage, but these viral-associated lesions were mild compared with coinfected piglets (Figure 2A). Three of the coinfected animals presented serositis (3/6), while serositis was completely absent in the Gps alone group. All control animals remained healthy throughout the experiment.

Lung scores were assigned according to the pathological degree of infected lung tissue. The mean lung scores (±SD) are shown in Figure 2B. There were significant statistical differences between the coinfected group and the Gps alone group (0.75 ± 0.35) (*p* < 0.05), while animals challenged with HP-PRRSV2 alone (4.50 ± 0.78) exhibited lung damage similar to that of the coinfected group (7.05 ± 0.61). However, the damage was more serious in coinfection with a significantly greater abnormal area than the HP-PRRSV2 infection alone (Figure 2A). Furthermore, there were no significant differences among the Gps and control groups, whereas both the Gps- and HP-PRRSV2-alone-infected animals exhibited significant differences in lung scores (*p* < 0.05; Figure 2B).

### 3.3. Pathogen Shedding and Load

**Figure 3:**
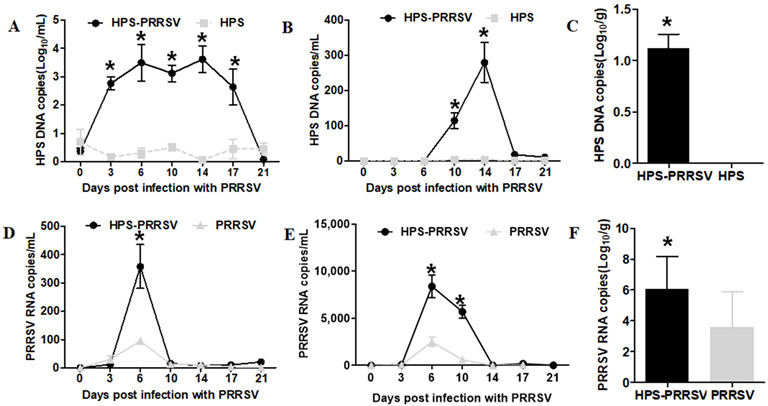
Pathogen load in nasal, blood, and lung samples from infected pigs. Gps and HP-PRRSV2 genome copy numbers were measured by qRT-PCR at 0, 3, 6, 10, 14, 17, and 21 dpi. (**A**,**D**) Nasal swabs and (**B**,**E**) blood samples were collected to determine mRNA copy numbers by qRT-PCR at various time points. Data are presented as the mean ± SD; * compared to single-infected groups within the same day (*p* < 0.05, Student’s *t*-test). (**C**,**F**) At 6 days post-HP-PRRSV2 challenge, piglet lung tissues were collected in each group and analyzed by qRT-PCR. The data are expressed as the mean logarithm of Gps or HP-PRRSV2 genomic copy number per gram (*n* = 3 in each group). * Significant differences compared with Gps or HP-PRRSV2 alone groups (*p* <0.05, Student’s *t*-test).

Coinfected pigs had higher Gps and HP-PRRSV2 titters in nasal swabs (Figure 3A,D). In coinfected piglets, the mean Gps titers in nasal swabs were significantly higher from 3 dpi and remained elevated until the end of the study (Figure 3A; *p* < 0.01). The load of HP-PRRSV2 in nasal swabs in coinfected piglets reached a maximum value on day 6 after HP-PRRSV2 infection, and the difference from the HP-PRRSV2 single-infection group was statistically significant (*p <* 0.05). At day 10 after HP-PRRSV2 infection, the load of HP-PRRSV2 in nasal swabs from the coinfected and HP-PRRSV2 single-infected groups decreased abruptly, with no statistical differences between the two groups (Figure 3D; *p* > 0.05).

The blood titers of Gps and HP-PRRSV2 are shown in Figure 3B,E. Gps was non-detectable in the Gps alone group, but it appeared in coinfected animals at 6 days after HP-PRRSV2 infection (peaked at 14 dpi), showing a significant difference (*p* < 0.01) from single-infected animals (Figure 3B). However, HP-PRRSV2 was detected in serum samples from both coinfected and HP-PRRSV2-alone-infected piglets at 3 days post-challenge (Figure 3E). The copy number of HP-PRRSV2 reached the maximum at 6 dpi in both groups, which was significantly higher (*p* < 0.05) in coinfected animals (Figure 3E).

Lung tissue samples were collected at 6 dpi to further determine the Gps and HP-PRRSV2 titers in single- and coinfected animals. The results were similar to those obtained for serum samples: Gps was undetectable in Gps single-infected animals, whereas coinfected animals had significantly higher mean genomic copies of Gps (Figure 3C; *p* < 0.01). HP-PRRSV2 was detected at a relatively high copy number in lung tissues from HP-PRRSV2-alone-infected piglets compared with coinfected animals (Figure 3F; *p* < 0.05). Together, these results are consistent with previous studies showing that Gps infection enhances HP-PRRSV2 replication [31]. This also validates our hypothesis that coinfection accelerates both HP-PRRSV2 and Gps replication in coinfected animals, causing greater damage than a single infection.

### 3.4. Serum Antibody Levels of Gps and HP-PRRSV2

**Figure 4:**
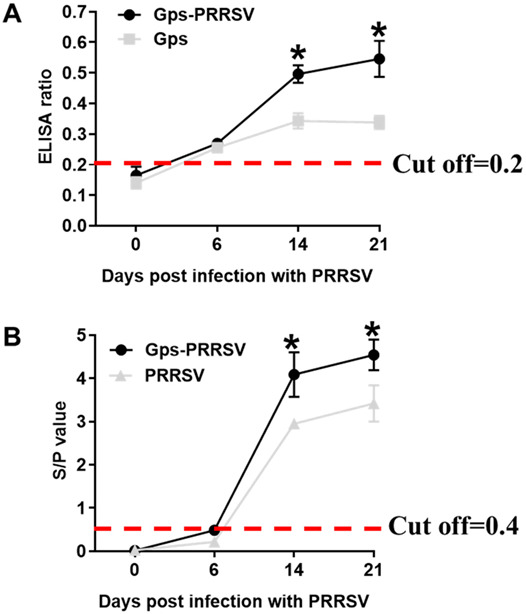
Antibody responses against Gps and HP-PRRSV2 infections. (**A**,**B**) Scheme 0 to 21 dpi and anti-Gps/HP-PRRSV2 antibodies were determined by ELISA. The horizontal line represents cut-off values for assays. Data are presented as the mean ± SD. * Compared with single-infected groups (Gps or HP-PRRSV2 alone) within the same day (*p* < 0.05, Student’s *t*-test).

Specific humoral immune response against HP-PRRSV2 and Gps in challenged animals is presented in Figure 4. At 0, 6, 14, and 21 days after HP-PRRSV2 infection, serum samples were determined for antibody levels by ELISA. The coinfected piglets produced specific antibodies against Gps, while animals infected with Gps alone did not exhibit a significant humoral response (Figure 4A). At 14 dpi, the coinfected animals produced much higher levels of Gps antibodies, which remained high (*p* < 0.05) until the end of the experiment (21 dpi; Figure 4A). By contrast, HP-PRRSV2 triggered antibody levels showed similar patterns in coinfected and HP-PRRSV2 alone groups (Figure 4B); however, S/P levels in coinfected animals were significantly higher than in HP-PRRSV2 single-infected animals at 14 and 21 dpi (Figure 4B). Consistent with a previous study in 2012 [31], these findings confirmed that HP-PRRSV2 infection enhanced Gps replication in coinfected animals, which promoted a strong antibody response against Gps.

### 3.5. Cytokine Analysis

**Figure 5:**
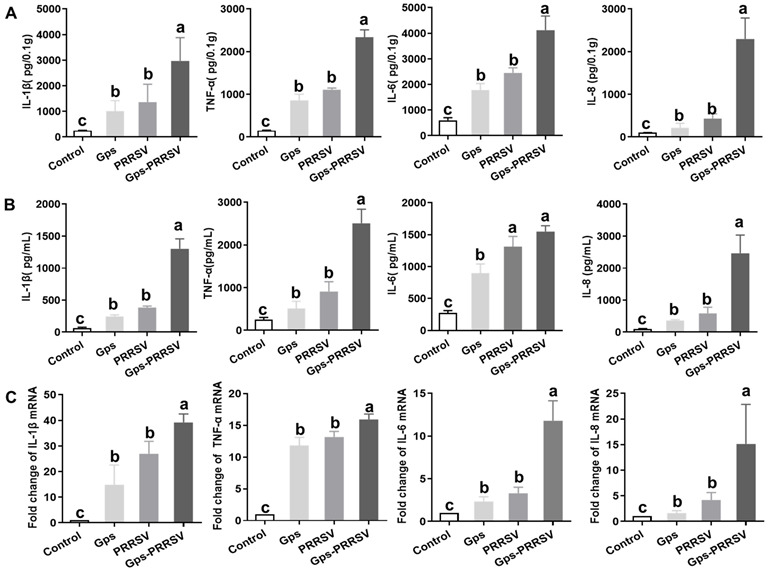
Proinflammatory cytokine levels in lungs and sera. At 6 days post-HP-PRRSV2 challenge, lung tissues and serum samples were collected from the pigs in each group. (**A**) Levels of IL-1β, TNF-α, IL-6, and IL-8 in lung homogenates were detected by ELISA. (**B**) Serum levels of IL-1β, TNF-α, IL-6, and IL-8 were detected by ELISA. (**C**) Cytokine mRNA levels in the lungs were determined by qRT-PCR. Data are the mean ± SD for three independent experiments (error bars). Data with different letters indicate significant differences at *p* < 0.05.

Microbial infection can change cytokine levels in animals [3,67]. Herein, we measured changes in the expression and abundance of cytokines TNF-ɑ, IL-1β, IL-8, and IL-6 at 6 dpi in lung tissue homogenates and serum by ELISA and qRT-PCR [70]. The levels of IL-1β, TNF-ɑ, and IL-8 were significantly higher (*p* < 0.05) in coinfected animals (up to 1500, 3000, and 3000 pg/mL, respectively), while both the single-infected animals had similar titers (*p* > 0.05; Figure 5A,B). Similar patterns were observed for TNF-ɑ and IL-6 in lung and serum samples. However, the IL-6 serum titer did not significantly differ between the HP-PRRSV2 single-infected and coinfected animals (Figure 5A,B).

Furthermore, there were no statistical differences in gene expression levels of IL-1β and TNF-ɑ among both types of single-challenged animals (Figure 5C), while IL-1β and TNF-α were upregulated in co-infected animals. In addition, the expression levels of IL-8 and IL-6 were much lower in both types of single-infected animals (Figure 5C). Interestingly, lung tissues from coinfected animals displayed an upregulation of IL-6 (12-fold) and IL-8 (14-fold) more than the single-infected groups (Figure 5C). The tested cytokines were statistically more upregulated in all infected animals than in control animals (Figure 5).

Concisely, in general, the maximum levels of cytokines were observed in coinfected animals at 6 dpi (Figure 5). Gps alone infection did not upregulate any of the investigated cytokines, while HP-PRRSV2 exposure following Gps inoculation stimulated the production of the same nature. These results are consistent with the localization of gross lung lesions: significant correlations were observed between pathological changes in the lungs and concentrations of TNF-ɑ, IL-1β, IL-8, and IL-6.

## 4. Discussion

Although single infections with bacteria or viruses alone can induce respiratory infections in pigs, coinfection with various pathogens is very common [28,71]. A Gps infection initiates an innate immune response and induces the production of inflammatory cytokines [72,73]. HP-PRRSV2 targets the immune system of pigs, impairing immune defense against pathogenic microbes and increasing host susceptibility to primary and secondary pathogens [64,74]. In this study, we found that HP-PRRSV2–Gps coinfection had aggravated clinical outcomes, increased pathogen shedding and load, and specific antibody and cytokine production at various time points following HP-PRRSV2 challenge after Gps infection. We observed that the coinfection enhanced the severity of the disease in different ways, including increased Gps and HP-PRRSV2 replication, which modulated the inflammatory response. In addition, pulmonary lesions in coinfected animals were more severe compared to those in single-infected animals.

Previous studies reported that piglets infected with HP-PRRSV2 alone or in combination with Gps suffered from severe interstitial pneumonia [7,74,75]. It is also suggested that secondary bacterial infection followed by HP-PRRSV2 infection exacerbates illness and mortality in infected piglets [76,77,78]. Here, we provided more comprehensive information on the effect of HP-PRRSV2 and Gps coinfection on respiratory disease pathogenicity in challenged piglets. We examined Gps and HP-PRRSV2 mRNA levels in nasal swabs, blood, and lung tissues at various time points of coinfection and single infection. The Gps load in nasal swabs was significantly higher from day 3 to 17 in coinfected animals compared with Gps single-infected animals (Figure 3A). Additionally, the serum Gps loads were significantly different between the coinfected and Gps single-infected animals at 10 and 14 days post-HP-PRRSV2 infection (Figure 3B). There was a significant increase in the Gps copy number in lung tissues of coinfected animals, while Gps was undetectable in Gps single-infected pigs (Figure 3C). From the above results, we concluded that HP-PRRSV2 infection promotes the proliferation of Gps in the nose, blood, and lungs.

Based on our in vivo findings, we concluded that Gps alone infection did not cause significant lesions in the lungs (Figure 2). Clinical signs and symptoms showed no rise in temperature, and even the post-mortem examination confirmed the same. This indicated that PPRSV2 infection enhances Gps colonization, which ultimately increases its copy number in the lung and blood (Figure 3). These in vivo findings are consistent with a previous report showing that Gps causes typical systemic polyserositis lesions only after coinfection with HP-PRRSV2 [78,79]. Notably, *Mycoplasma hyrhinis* coinfection with HP-PRRSV2 can also enhance pathological lesions in the lungs [80], which is consistent with our results showing that Gps coinfection with HP-PRRSV2 leads to lung consolidation and severe interstitial pneumonia.

We further investigated whether Gps infection affects HP-PRRSV2 pathogenicity. HP-PRRSV2 proliferation in nasal mucosa takes a very short time. There were significant differences of PRRSV load in the nasal mucosa between coinfection and HP-PRRSV2 single-infection groups at 6 dpi (Figure 3A). Moreover, HP-PRRSV2 copy numbers (400) were significantly higher in the coinfected animal blood and lung tissues (Figure 3B,C). From the above results, we concluded that Gps infection has a significant impact on HP-PRRSV2 pathogenicity. Additionally, lung consolidation and severe interstitial pneumonia were observed in coinfected animals (Figure 2). Enhanced HP-PRRSV2 replication in coinfected animals led to more severe clinical outcomes, involving earlier and greater immune and inflammatory responses (Figure 4 and Figure 5). This conclusion is consistent with the study of Yu et al. in 2012 [31].

How the coinfection of viruses and bacteria enhances disease severity is not fully understood [81]. Coinfection often upregulates various cytokines that help microbes with replication, but causes detrimental damage to infected tissues [3,82]. HP-PRRSV2 infection has several mechanisms that make infected animals prone to secondary bacterial infection, including increased expression of cellular receptors that enhance colonization and modification of host immune responses [7,83,84]. Here, we found that the production of inflammatory cytokines (TNF-ɑ, IL-1β, IL-8, and IL-6) was significantly increased in coinfected animals compared with both types of single-infected pigs (Figure 5). This is consistent with a previous study showing that PRRSV infection upregulated cytokines (TNF-ɑ, IL-1β, IL-8, and IL-6) in pig sera and promoted the bacterial load of 11 bacterial species in the lungs, including Gps (Li et al., 2017). In this study, coinfection upregulated inflammatory cytokine in lung tissue and blood as well (Figure 5), but the exact molecular mechanism is unknown.

HP-PRRSV2 and Gps are known to stimulate each other’s replication when coinfected, but the impacts of coinfection on clinical outcomes, the kinetics of the immune system and inflammatory responses, and pathogen load and shedding in piglets remain poorly understood [7,31]. Herein, piglets challenged with HP-PRRSV2 alone displayed significant differences in cytokine production, lung score, and macroscopic lesions in the lungs compared with coinfected animals (Figure 2 and Figure 5). The mRNA levels and antibody titer results showed that both HP-PRRSV2 and Gps replicated more rapidly and elicited more severe local inflammatory responses in coinfected animals than in any of the single-infected animals (Figure 3 and Figure 4). A similar trend has been reported for SIV and Gps coinfection [67]. The significant influence of both pathogens on the systemic inflammatory response was only present in coinfected animals (Figure 5). These outcomes are consistent with the fact that PRRSV and S. suis serotype 2 coinfection upregulated TNF-α, IL-1β, IL-8, and IL-6 gene expressions, compared with single-infected animals [7]. Nonetheless, further studies are needed to elucidate the molecular mechanism by which HP-PRRSV2–Gps coinfection enhances the production of pro-inflammatory cytokines increasing the disease severity.

## 5. Conclusions

There were lung lesions among all of the challenged piglets. HP-PRRSV2 infection potentiates the degree of lung lesions and facilitates Gps replication in porcine lungs. Gps infection enhances the copy number of HP-PRRSV2 in nasal shedding, blood, and lung tissues. Enhanced Gps and HP-PRRSV2 replication and stronger systemic/local inflammatory responses aggravate clinical signs in coinfected piglets. The coinfected piglets had a more severe fever, pathological changes, microscopic lung lesions, and a higher pathogen load than any of the single-infected piglets. These results showed that the secondary infection of HP-PRRSV2 will aggravate lung disease and chronic inflammation in the case of common Gps infection of the upper respiratory tract in piglets. Therefore, it is necessary to prevent and control HP-PRRSV2 infection in such cases.

## Figures and Tables

**Table 1:** Scoring criteria for clinical symptoms of experimental piglets.

Scoring Criteria	Respiratory Signs	Nasal Discharge, Coughing, Anorexia, and Sneezing	Temperature	Lung Lesions
0	normal (<34 breaths/min)	absent	normal	no lesions
1	slightly elevated (35–40 breaths/min)	present	rectal temperature exceeded 40 °C	lesions affecting <25% of the lobe surface
2	moderately elevated (41–45 breaths/min with slight abdominal breathing)	/	/	lesions affecting 25–49% of the lobe surface
3	elevated (>46 breaths/min with distinct abdominal breathing)	/	/	lesions affecting 50–74% of the lobe surface
4	/	/	/	lesions affecting >75% of the lobe surface

“/” means that the standard does not have this score.

## Data Availability

Not applicable.
